# Large-Scale No-Show Patterns and Distributions for Clinic Operational Research

**DOI:** 10.3390/healthcare4010015

**Published:** 2016-02-16

**Authors:** Michael L. Davies, Rachel M. Goffman, Jerrold H. May, Robert J. Monte, Keri L. Rodriguez, Youxu C. Tjader, Dominic L. Vargas

**Affiliations:** 1 Access and Clinic Administration Program (ACAP), U.S. Department of Veterans Affairs, Washington, DC 57741, USA; michael.davies@va.gov; 2 Veterans Engineering Resource Center, VA Pittsburgh Healthcare System, Pittsburgh, PA 15240, USA; rachel.goffman@va.gov (R.M.G.); robert.monte@va.gov (R.J.M.); keri.rodriguez@va.gov (K.L.R.); 3 Joseph M. Katz Graduate School of Business, University of Pittsburgh, Pittsburgh, PA 15260, USA; jerrymay@katz.pitt.edu (J.H.M.); yotst1@katz.pitt.edu (Y.C.T.); 4 Center for Health Equity Research and Promotion, VA Pittsburgh Healthcare System, Pittsburgh, PA 15240, USA; 5 Department of Medicine, Division of General Internal Medicine, University of Pittsburgh School of Medicine, Pittsburgh, PA 15261, USA

**Keywords:** outpatient appointments, no-shows, frequent attenders, statistical analysis

## Abstract

Patient no-shows for scheduled primary care appointments are common. Unused appointment slots reduce patient quality of care, access to services and provider productivity while increasing loss to follow-up and medical costs. This paper describes patterns of no-show variation by patient age, gender, appointment age, and type of appointment request for six individual service lines in the United States Veterans Health Administration (VHA). This retrospective observational descriptive project examined 25,050,479 VHA appointments contained in individual-level records for eight years (FY07-FY14) for 555,183 patients. Multifactor analysis of variance (ANOVA) was performed, with no-show rate as the dependent variable, and gender, age group, appointment age, new patient status, and service line as factors. The analyses revealed that males had higher no-show rates than females to age 65, at which point males and females exhibited similar rates. The average no-show rates decreased with age until 75–79, whereupon rates increased. As appointment age increased, males and new patients had increasing no-show rates. Younger patients are especially prone to no-show as appointment age increases. These findings provide novel information to healthcare practitioners and management scientists to more accurately characterize no-show and attendance rates and the impact of certain patient factors. Future general population data could determine whether findings from VHA data generalize to others.

## 1. Introduction

No-show patient appointments have been defined as “patients who neither kept nor cancelled scheduled appointments” [1]. Although the documented rates of missed appointments may vary somewhat between countries, health care systems, and clinical settings [2], appointment-breaking behaviors constitute a widespread, global issue [2,3,4]. No-show rates have been shown to range from 15%–30% in general medicine clinics and urban community centers [5,6]. It has also been reported that no-show rates can reach as high as 50% in primary care [7].

Missed appointments represent a major burden on health care systems and have a negative impact on patient care [8]. For example, patient no-shows can cause scheduling and operational difficulties for clinics [9,10] and reduced productivity [11,12]. No-shows can also reduce access to care [13], as well as interrupt continuity of care and effective disease management for patients [12,13,14,15,16]. Although the level of economic impact differs according to the health care system, the overall financial cost of no-shows is substantial [17]. In one of the few national studies, it was estimated that the financial cost to the United Kingdom National Health Service (from nonattendance at outpatient clinics) was approximately ₤790 million per year [3]. 

The Veterans Health Administration (VHA), one of the largest integrated healthcare delivery systems in the United States [18], has had a similar experience. For example, it was projected that approximately 18 percent of the scheduled annual VHA outpatient appointments (in 10 performance measure clinics) were unused in fiscal year 2008. The VHA estimates the cost of no-shows and unused appointments is approximately $564 million annually [17]. To reduce missed clinic appointments, organizations must mine data [19], develop effective appointment-keeping strategies [20,21], and systemically implement strategies in their operations [22].

Attempts to do simulation modeling using estimates of no-show and frequent attendance rates, distributions, and patient factor interactions are often confounded by (1) study precision and validity; (2) non-generalizable results from relatively small, unique, and poorly sampled studies; (3) pervasive difficulties with bias; (4) non-standardized problem definitions and methodologies; and (5) inconsistent statistical reporting of results [11,23] Indeed, computer modeling that ignores realistic conditions, parameters, and thresholds are often found to perform poorly [24]. Therefore, improving access to unbiased information derived from large sets of data is essential in order to address these problems [10,25]. 

Studies identifying predictors of appointment no-shows are important to clinicians. Factors that predict no-show allow researchers and clinicians to improve performance. For example, appointment overbooks may contribute to unnecessary waiting times for patients and overtime for practitioners. However, better forecasting of attendance rates can minimize these errors and increase access to quality healthcare [26].

The Pittsburgh Veterans Engineering Resource Center (VERC) led a VHA effort known as the National Initiative to Reduce Missed Opportunities (NIRMO). This initiative uses predictive and simulation modeling, data mining and statistical analysis on large data sets to design and test techniques to reduce patient appointment breaking behaviors. The NIRMO work is important because it reveals the impact of universal factors from a broad segment of patients accessing all types of care at multiple sites over many years [27,28,29,30,31,32].

This report is descriptive, retrospective and observational from VHA patient-level appointment data over from fiscal year 2007 to 2014. It describes how no-show rates vary with patient age, gender, and with appointment age.

## 2. Materials and Methods

### 2.1. Data Sources

The data for this descriptive, retrospective project were extracted from the United States Department of Veterans Affairs Electronic Health Record (EHR) system, known as the Veterans Health Information Systems and Technology Architecture (VistA). Appointments were categorized into two types: complete and incomplete. A complete appointment describes any appointment that was scheduled and completed, while an incomplete appointment was one that was scheduled but the patient was not seen by a healthcare provider and it was not cancelled in advance.

### 2.2. Data Collection and Cleaning

All scheduled outpatient appointments were examined for eight years (FY2007 through FY2014) from three large, tertiary level VHA facilities from three geographically different locations: Pittsburgh, Tampa, and Houston. The VHA outpatient appointments were grouped into stop codes and then aggregated into six service lines: Primary Care; Mental Health; Specialty Medicine; Rehabilitation; Surgery; and Other. For a general description of service line departments, please see Table A1 in the appendix.

The available data fields included a de-identified patient ID number, patient age at the time of the appointment, gender, the date the appointment was entered into the system, the date and time for which the appointment was scheduled, and the final appointment status (complete or incomplete). Additionally patients were classified into new or already established for a given appointment. New patients were those who did not complete an appointment within a given clinic type during the prior 24 months. Some patients were active in the VA outpatient system for all years studied, while others were active for only a subset of those years. The appointment age was determined by calculating the number of days between the date the appointment was created and the date the appointment was completed. The final data set included 555,183 patients, who scheduled 25,050,479 appointments.

### 2.3. Data Analyses and Methods

Multifactor analysis of variance (ANOVA) was performed, with no-show rate as the dependent variable and the following factors; A. New *versus* Established patients; B. Appointment Age Groups; C. Patient Age Groups; D. Gender; E. Service Line. Pairwise comparisons were performed using 95% Tukey intervals (other interval types gave similar results). All interactions of order two were included to uncover the significance of the main effects on the no-show rate. This allowed for a more detailed look at the interactions between gender, appointment and patient age, and whether the patient was new or established. Service line was included in the analysis to factor in differences in scheduling.

## 3. Results and Discussion

The following section describes the analysis and discusses the results of the subsequent ANOVA. Table 1 shows that the main effects and all the interactions of order 2 are significant. Note that the interaction between Appointment Age Group and Gender has the smallest significance as indicated by the F-Ratio in Table 1.

**Table 1:** Analysis of variance results for no-show rate—Type III sum of squares.

Main Effects	Sum of Squares	Df	Mean Square	*f*-ratio	*p*-value
A: New *vs* Est	11679.9	1	11679.9	96782.68	0
B: Appt Age Group	14534.5	9	1614.94	13381.88	0
C: Age Group	10367.9	13	797.527	6608.54	0
D: Gender	10.5457	1	10.5457	87.38	0
E: Service Line	1254.31	5	250.682	2078.72	0
**Interactions**	**Sum of Squares**	**Df**	**Mean Square**	***f*-ratio**	***p*-value**
AB	14906.3	9	1656.25	13724.16	0
AC	246.517	13	18.9628	157.13	0
AD	13.2343	1	13.2343	109.66	0
AE	4289.99	5	857.997	7109.61	0
BC	11011.8	117	94.1178	779.89	0
BD	59.6455	9	6.62728	54.92	0
BE	6113.94	45	135.865	1125.82	0
CD	302.393	13	23.261	192.75	0
CE	2057.52	65	31.6541	262.29	0
DE	66.027	5	13.2054	109.42	0
Residual	3.02 × 10^6^	25048804	0.120681		
Total (Corrected)	3.21 × 10^6^	25049115			

All *f*-ratios are based on the residual mean square error.

### 3.1. Gender and Age Frequencies

The VHA population was predominately male (91.47%) and between 60 and 70 years of age or older (29.32% of the total population). Table 2 provides a breakdown of 14 age groupings compared to the total population. This sample contains about 10 times more males than females. The highest female appointment frequency occurs at age 45 to 64 compared to males at age 55 to 69. This pattern reflects recent national trends of more women in the military.

**Table 2:** Appointment frequencies for males and females.

Age Group	Females		Males	
	Number of Appointments	% of Total	Number of Appointments	% of Total
0–24	19,673	0.92%	63,943	0.28%
24–29	131,159	6.14%	494,210	2.16%
30–34	173,758	8.14%	583,566	2.55%
35–39	147,651	6.91%	423,802	1.85%
40–44	199,126	9.32%	697,399	3.04%
45–49	241,899	11.33%	923,322	4.03%
50–54	328,301	15.37%	1,599,931	6.98%
55–59	339,660	15.91%	2,339,433	10.21%
60–64	277,320	12.99%	4,378,852	19.11%
65–69	124,670	5.84%	4,311,926	18.82%
70–74	42,317	1.98%	2,001,707	8.74%
75–79	32,224	1.51%	1,746,780	7.62%
80–84	24,441	1.14%	1,575,799	6.88%
85+	52,891	2.48%	1,773,356	7.74%
Total	2,135,090		22,914,026	

**Figure 1:**
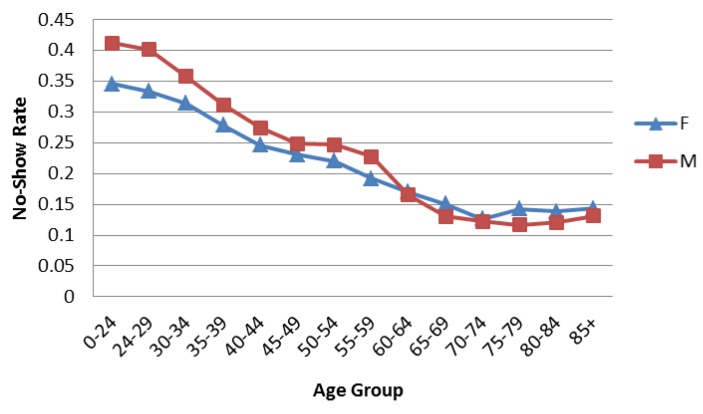
No-show rates by age group for all service lines.

**Figure 2:**
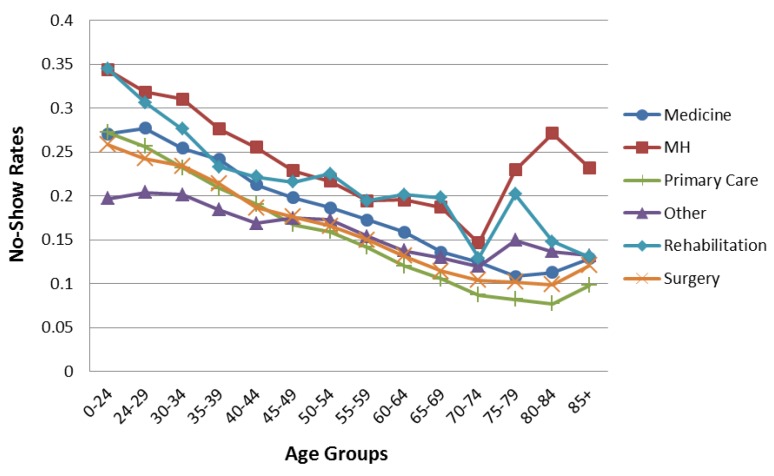
No-show rates for female patients by age groups separated by service line.

**Figure 3:**
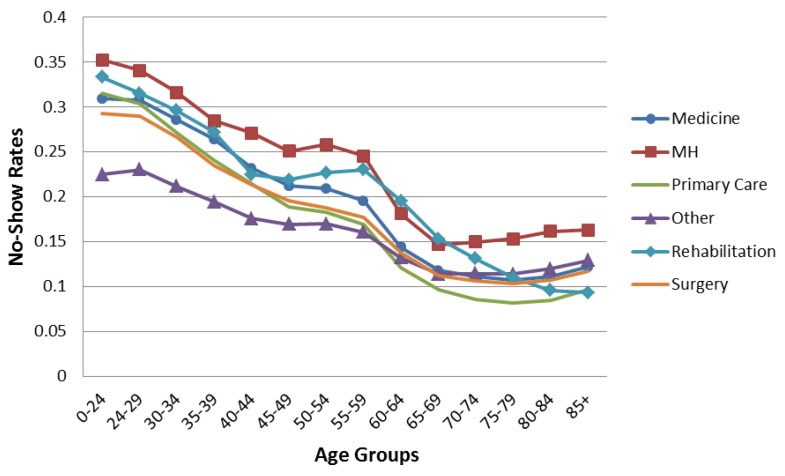
No-show rates for male patients by age groups separated by service line.

Figure 1 shows the aggregated no-show data trends. The overall average no-show rates decrease with age until 75–79, when they increase slightly. Males have higher no-show rates than females until age 65, when males and females exhibit similar rates. Figure 2 and Figure 3 compare the overall pattern segmented by service line, gender, and age. The Medical, Primary Care, and Surgery Service Lines have patterns similar to the overall results in Figure 1. Interestingly, Mental Health and Rehabilitation reveal females above age 74 with higher than overall expected no-show rates. While this observation could be influenced by relatively small numbers (see Table 2), it is an area for further study. Appointment frequencies are broken down, in Table 3, to reflect the total number of appointments for each service line by gender and age grouping.

### 3.2. Appointment Age

**Figure 4:**
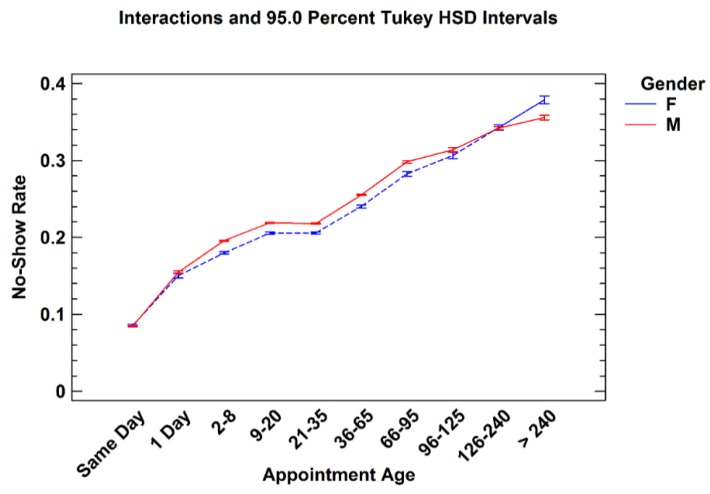
No-show rates for males and females by appointment age groups.

Appointment age is defined as the difference between the date an appointment was scheduled and the future pending appointment date. This shows “how far in advance” an appointment is created or made. Consistent with past research, no-show rates increase as appointment age increases [32,33]. While no-show rates for males were generally higher than females, males and females tended to have similar rates with respect to appointment age, shown in Figure 4. The appointment frequencies, in Table 4, show that the majority of the total appointments (63.6%) occurred between two and 65 days of lead time. There were also a large number of same day appointments. This holds true across all service line, shown in Table 5.

**Table 3:** Appointment frequencies for all service lines.

Age Group	Medicine		Mental Health		Primary Care		Other		Rehabilitation		Surgery	
	Female	Male	Female	Male	Female	Male	Female	Male	Female	Male	Female	Male
0–24	1754	6586	3018	11,944	4321	11,381	7542	21,041	1112	7302	1926	5689
24–29	12,304	45,206	20,822	101,700	29,131	89,165	46,589	170,995	5696	47,220	16,617	39,924
30–34	17,149	57,504	29,122	126,908	36,642	107,362	60,358	197,751	7532	41,984	22,955	52,057
35–39	16,136	44,540	26,023	83,089	29,326	81,285	49,719	144,080	6533	28,317	19,914	42,491
40–44	22,920	81,845	33,637	134,158	37,011	132,791	68,282	229,308	9208	41,837	28,068	77,460
45–49	27,562	113,670	41,140	167,283	44,297	178,699	83,010	299,944	11,078	51,745	34,812	111,981
50–54	39,846	199,999	51,901	284,787	61,392	301,947	112,290	532,357	15,846	77,383	47,026	203,458
55–59	43,107	326,969	47,007	352,886	64,995	444,169	118,507	787,947	16,129	106,456	49,915	321,006
60–64	36,171	706,403	31,860	587,188	58,037	832,968	96,595	1,416,052	13,284	196,063	41,373	640,178
65–69	18,416	753,579	10,289	394,470	26,057	849,214	43,823	1,416,132	6076	203,456	20,009	695,075
70–74	6624	360,514	2256	89,101	8834	427,438	15,107	675,099	1817	106,472	7679	343,083
75–79	5765	295,694	1372	47,478	6874	398,430	10,945	599,550	1493	107,521	5775	298,107
80–84	3440	239,636	1327	49,929	5168	369,757	8965	530,041	1219	121,908	4322	264,528
85+	7613	244,755	1991	52,690	12,754	423,617	18,113	587,055	4364	171,569	8056	293,670

**Table 4:** Appointment frequencies and percentages by gender for appointment age groups.

Appt Age (Day)	Female		Male		Total	
	Number of Appts	% Total of Female Appts	Number of Appts	% Total of Male Appts	Number of Appts	% of Total Appts
Same Day	395,364	18.51%	3,689,640	16.1%	4,085,004	16.31%
1	84,264	3.95%	814,249	3.55%	898,513	3.59%
2–8	313,590	14.68%	3,128,648	13.65%	3,442,238	13.74%
9–20	364,528	17.07%	3,842,965	16.77%	4,207,493	16.8%
21–35	417,340	19.54%	4,686,005	20.45%	5,103,345	20.37%
36–65	268,719	12.58%	2,906,762	12.69%	3,175,481	12.68%
66–95	113,174	5.3%	1,258,943	5.49%	1,372,117	5.48%
96–125	54,656	2.56%	726,417	3.17%	781,073	3.12%
126–240	86,278	4.04%	1,364,690	5.96%	1,450,968	5.79%
>240	37,560	1.76%	496,253	2.17%	533,813	2.13%
Total	2,135,473		22,914,572		25,050,045	

**Table 5:** Appointment frequencies and percentages for all service lines by appointment age groups.

Appt Age (Day)	Medicine		Mental Health		Other		Primary Care		Rehabilitation		Surgery		Total	
	Number of Appts	% Total Medicine appts	Number of Appts	% Total Number of MH Appts	Number of Appts	% Total Number of Other Appts	Number of Appts	% Total Number of PC Appts	Number of Appts	% Total Number of Rehab Appts	Number of Appts	% Total Number of Surgery Appts	Number of Appts	% Total Number of All Appts
Same Day	441,431	11.8%	413,203	14.8%	1,878,555	22.5%	717,933	14.2%	262,853	18.6%	371,093	10.0%	4,085,068	16.3%
1	111,423	3.0%	86,504	3.1%	297,810	3.6%	202,417	4.0%	73,192	5.2%	127,180	3.4%	898,526	3.6%
2–8	472,527	12.6%	449,746	16.1%	1,038,066	12.4%	641,199	12.6%	299,801	21.3%	540,970	14.6%	3,442,309	13.7%
9–20	664,157	17.8%	511,787	18.4%	1,271,704	15.2%	762,196	15.0%	307,757	21.8%	689,969	18.7%	4,207,570	16.8%
21–35	857,743	23.0%	566,629	20.3%	1,393,564	16.7%	1,124,022	22.2%	302,548	21.4%	858,938	23.2%	5,103,444	20.4%
36–65	571,111	15.3%	441,443	15.8%	838,245	10.0%	690,110	13.6%	127,235	9.0%	507,387	13.7%	3,175,531	12.7%
66–95	229,025	6.1%	190,068	6.8%	422,612	5.1%	257,315	5.1%	24,462	1.7%	248,664	6.7%	1,372,146	5.5%
96–125	141,963	3.8%	80,148	2.9%	253,607	3.0%	164,523	3.2%	6,332	0.4%	134,508	3.6%	781,081	3.1%
126–240	201,667	5.4%	43,991	1.6%	658,778	7.9%	380,146	7.5%	3,858	0.3%	162,545	4.4%	1,450,985	5.8%
>240	44,785	1.2%	1,925	0.1%	295,111	3.5%	133,339	2.6%	2,681	0.2%	55,978	1.5%	533,819	2.1%
Total	3,735,832		2,785,444		8,348,052		5,073,200		1,410,719		3,697,232		25,050,479	

#### 3.2.1. New *versus* Established Patients

**Figure 5:**
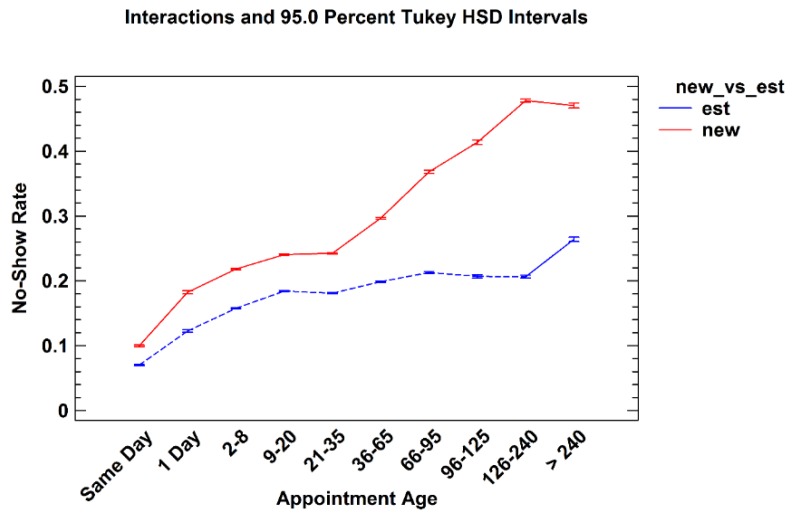
No-show rates for new and established patients by appointment age groups.

New patients are defined as those who did not have a completed appointment within a single service line clinic during the prior 24 months. Figure 5 shows the no-show rates for both new and established patients based on their appointment age. While there was only a slight difference found between rates for same day appointments, nevertheless there were significant differences between new and established patients across all appointment age groups. It is hypothesized that this finding reflects a new patient’s desire to address their clinical needs quickly. This data suggests clinic managers and practices should be particularly attentive to their new patient waiting times.

#### 3.2.2. Patient Age *vs.* Appointment Age

**Figure 6:**
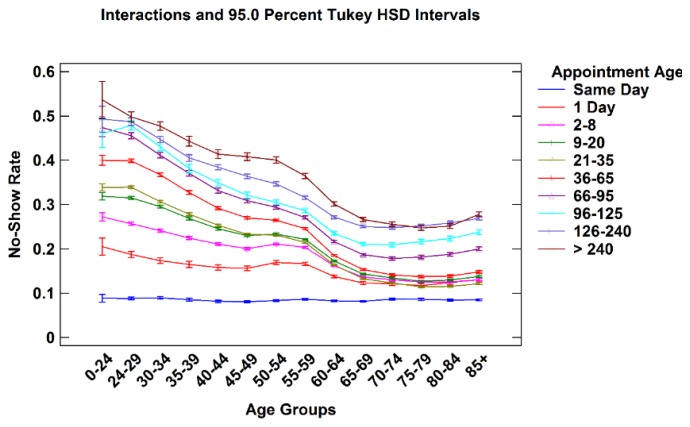
No-show rates for all appointment age groups by patient age.

Figure 6 shows an interaction plot of patient age and appointment age. Each line represents appointments that fall into one of nine progressively longer appointment age time groups displayed by patient age. While overall no-show rates increase with appointment age for all patient age groups, the relative impact as shown by the shape of the lines, are different. Same Day appointments generate a relatively constant no-show rate across all patient age groups. However, as appointment age increases, younger patient’s no-show rates dramatically increase compared to older patients. In addition, appointment age of even one to eight days is disproportionally higher in younger patients compared to older patients. As patient age increases, the overall pattern seen in Figure 1, Figure 2 and Figure 3 emerges. This observation suggests that managers may consider confirming the intention to keep appointments especially for young patients with longer appointment ages.

## 4. Conclusions

This paper describes the variation of no-show rates with patient age, gender, appointment age, and type of request within six individual service line of the United States VHA. The analyses revealed that males had higher no-show rates than females to age 65 where males and females exhibited similar rates. Average no-show rates decrease with age until age 75–79, whereupon they increase. No-show rates increase as appointment age lengthens for all age groups. Younger patients are especially prone to no-show as appointment age increases. New patients no-show at higher rates than established patients, especially beyond 36 days of lead time. These findings suggest particular attention to female patients over age 74 in Mental Health and Rehabilitation may be warranted.

This data has several limitations. The VA population does not map directly to the general United States patient population due to the greater percentages of older, male Veterans and the fact that female Veterans tend to be younger than male Veterans [34]. This data is consistent with the overall VA population median age of 57 years and approximately 90% male. Likewise, the age distribution for females is skewed more heavily towards lower age groupings as in the overall VA population [29]. Further analyses may determine if the findings are present in a non-VA population. While this data is available by service line or type, the study did not include diagnosis-specific information. For that reason, there may be additional diagnosis-related factors influencing patient appointment attendance behaviors that influence these results. 

Many additional factors have been associated with nonattendance. These factors include a patient’s race and ethnicity, socio-economic status, marital status, beliefs about their symptoms, source of illness, and severity of the patient’s condition [16,25,26,27,35,36]. Further, no-shows have been associated with structural barriers, such as distance to the clinic and lack of transportation [25,37]. Additionally, patient no-shows have been shown to vary by physician characteristics, patient-physician interaction, clinic access, administrative processes, and environmental factors including team communication and on-time appointments [25]. VHA data reveals no-show variation by geographical region and rural and urban settings. To best predict and minimize no-show rates, the influence of these additional factors may be important to understand and manage.

These findings from VHA longitudinal data allow understanding of no-shows from a large and statistically significant multi-year data set with little sampling bias. Practitioners working in the areas of operational research may find the results useful in order to more accurately characterize no-show and frequent attendance rates, and patient factor interactions. As a result of this study, clinicians and managers may wish to focus special attention on young male patients, new patients, and females over 74 in Mental Health and Rehabilitation. Computer and analytical modeling, as well as scheduling system re-engineering, may use this information to answer important questions regarding patient appointment behavior predictions and profiles. Future examination of data from the general population is needed to determine if the findings are generalizable beyond this population.

## Appendix

**Table A1:** Service line details.

Medicine	Nutrition/Dietetics	Radiation Therapy	General Internal Medicine	Allergy Immunology
		Cardiology	Dermatology	Endocrinology
		Diabetes	Gastroenterology	Hematology
		Oncology	Hypertension	Infectious disease
		Pacemaker	Pulmonary	Renal
		Rheumatology	Neurology	Oncology
		Anti-Coagulation Clinic	Geriatrics	Alzheimer's/Dementia Clinic
		Endoscopy	Chemotherapy	Cardiac Catheterization
		Cardiac Stress Test	Hepatology Clinic	Sleep Medicine
Mental Health	Mental Health Clinic	Psychiatry	Psychology	Substance Use Disorder
Other	Pulmonary Function	X-ray	EEG	EKG
		Laboratory	Nuclear Medicine	Ultrasound
		Respiratory Therapy	Home Treatment/Services	Health Screening
		Residential Care	Social Work Service	Topography
		Emergency Dept	Urgent Care Unit	Sleep Study
		Computerized Tomography	MRI	Interventional Radiography
		Magnetoencephalography	Brachytherapy Treatment	Alternative Medicine
		Clinical Pharmacy	Dental	Care/Case Manager
		Recreation Therapy Service	Blind Rehab	Parkinson's Disease Service
		Obstetrics	Genomic Care	Pediatrics
		Family Practice	Epilepsy	ALSs Center
		Hospice Care	Amputation Clinic	Cast clinic
		Chiropractic Care	Low Vision care	Transplant
		Research	Bronchoscopy	Hemodialysis
Primary Care	Primary Care/Medicine			
Rehabilitation	Enterostomal Care	Radionuclide Therapy	Polytrauma	Physical Medicine Audiology
		Speech-Language Pathology	Physical Therapy	Occupational Therapy
		Spinal Cord Injury	Electromyogram	Kinesiotherapy
		Intermed Low Vision Care		
Surgery	General Surgery	Cardiac Surgery	ENT	Gynecology
		Hand surgery	Neurosurgery	Ophthalmology
		Optometry	Orthopedics	Plastic surgery
		Podiatry	Proctology	Thoracic Surgery
		Urology	Vascular Surgery	Dialysis
		Prosthetics/Orthotics	Anesthesia Surgical Consult	Pain Clinic

## References

[1] Tuso P.J., Murtishaw K., Tadros W. (1999). The easy access program: A way to reduce patient no-show rate, decrease add-ons to primary care schedules, and improve patient satisfaction. Perm. J..

[2] Perron N.J., Dao M.D., Kossovsky M.P., Miserez V., Chuard C., Calmy A., Gaspoz J.M. (2010). Reduction of missed appointments at an urban primary care clinic: A randomised controlled study. BMC Fam. Pract..

[3] Atun A.R., Sittampalam S.R., Mohan A. (2005). Uses and Benefits of SMS in Healthcare Delivery.

[4] Turkcan A., Nuti L., DeLaurentis P.C., Tian Z., Daggy J., Zhang L., Lawley M., Sands L., Denton B.T. (2013). No-show modeling for adult ambulatory clinics. Handbook of Healthcare Operations Management.

[5] Goldman L., Freidin R., Cook E.F., Eigner J., Grich P. (1982). A multivariate approach to the prediction of no-show behavior in a primary care center. Arch. Intern. Med..

[6] Hixon A.L., Chapman R.W., Nuovo J. (1999). Failure to keep clinic appointments: Implications for residency education and productivity. Fam. Med..

[7] Lacy N.L., Paulman A., Reute M.D., Lovejoy B. (2004). Why we don’t come: Patient perceptions of no-shows. Ann. Fam. Med..

[8] Moore C.G., Wilson-Witherspoon P., Probst J.C. (2001). Time and money: Effects of no-shows at a family practice residency clinic. Fam. Med..

[9] Gupta D., Denton B. (2008). Appointment scheduling in health care: Challenges and opportunities. IIE Trans..

[10] Kopach R., DeLaurentis P.C., Lawley M., Muthuraman K., Ozen L., Rardin R., Wan H., Intrevado P., Qu X., Willis D. (2007). Effects of clinical characteristics on successful open access scheduling. Health Care Manag. Sci..

[11] Daggy J., Lawley M., Willis D., Thayer D., Suelzer C., DeLaurentis P.C., Turkcan A., Chakraborty S., Sands L. (2010). Using no-show modeling to improve clinic performance. Health Inform. J..

[12] Nguyen D.L., DeJesus R.S., Wieland M.L. (2011). Missed appointments in resident continuity clinic: Patient characteristics and health care outcomes. J. Grad. Med. Educ..

[13] Johnson B.J., Mold J.W., Pontius J.M. (2007). Reduction and management of no-shows by family medicine residency practice exemplars. Ann. Fam. Med..

[14] Bowser D.M., Utz S., Glick D., Harmon R. (2010). A systematic review of the relationship of diabetes mellitus, depression, and missed appointments in a low-income uninsured population. Arch. Psychiatr. Nurs..

[15] Nuti L.A., Lawley M., Turkcan A., Tian Z., Zhang L., Chang K., Willis D.R., Sands L.P. (2012). No-shows to primary care appointments: Subsequent acute care utilization among diabetic patients. BMC Health Serv. Res..

[16] Schectman J.M., Schorling J., Voss J.D. (2008). Appointment adherence and disparities in outcomes among patients with diabetes. J. Gen. Intern. Med..

[17] United States Department of Veterans Affairs (2008). Audit of Veterans Health Administration's Efforts to Reduce Unused Outpatient Appointments.

[18] United States Department of Veterans Affairs (2007). Focusing on the Nation’s Priorities.

[19] D’Avolio L.W. (2009). Electronic Medical Records at a crossroads: Impetus for change or missed opportunity?. JAMA.

[20] Perron N.J., Dao M.D., Righini N.C., Humair J.P., Broers B., Narring F., Haller D.M., Gaspoz J.M. (2013). Text-messaging *versus* telephone reminders to reduce missed appointments in an academic primary care clinic: A randomized controlled trial. BMC Health Serv. Res..

[21] Parikh A., Gupta K., Wilson A.C., Fields K., Cosgrove N.M., Kostis J.B. (2010). The effectiveness of outpatient appointment reminder systems in reducing no-show rates. Am. J. Med..

[22] Woodcock E.W. (2007). Mastering Patient Flow: Using Lean Thinking to Improve Your Practice Operations.

[23] Liu N., Ziya S., Kulkarni V.G. (2010). Dynamic scheduling of outpatient appointments under patient no-shows and cancellations. Manuf. Serv. Oper. Manag..

[24] Luo J., Kulkarni V.G., Ziya S. (2012). Appointment scheduling under patient no-shows and service interruptions. Manuf. Serv. Oper. Manag..

[25] George A., Rubin G. (2003). Non-attendance in general practice: A systematic review and its implications for access to primary health care. Fam Pract..

[26] Samorani M., LaGanga L. (2015). Outpatient appointment scheduling given individual day-dependent no-show predictions. Eur. J. Oper. Res..

[27] Bagalman E. (2013). Suicide Prevention Efforts of the Veterans Health Administration.

[28] Mitchell A.J., Selems T. (2007). Why don’t patients attend their appointments? Maintaining engagement with psychiatric services. Adv. Psychiatr. Treat..

[29] United States Department of Veterans Affairs (2011). Interventions to Improve Veteran’s Access to Care: A Systematic Review of the Literature.

[30] Weinberger M., Oddone E.Z., Henderson W.G. (1996). Does increased access to primary care reduce hospital readmissions? Veterans Affairs Cooperative Study Group on Primary Care and Hospital Readmission. N. Engl. J. Med..

[31] Gardner L.I., Marks G., Craw J.A., Wilson T.E., Drainoni M.L., Moore R.D., Mugavero M.J., Rodriguez A.E., Bradley-Springer L.A., Holman S. (2012). Retention in Care Study Group. A low-effort, clinic-wide intervention improves attendance for HIV primary care. Clin. Infect. Dis..

[32] Kirsh S.R., Lawrence R.H., Aron D.C. (2008). Tailoring an intervention to the context and system redesign related to the intervention: A case study of implementing shared medical appointments for diabetes. Implement. Sci..

[33] Giachetti R.E. A simulation study of interventions to reduce appointment lead-time and patient no-show rate. Proceedings of the 2008 Winter Simulation Conference.

[34] Kessler C.S., Bhandarkar S., Casey P., Tenner A. (2011). Predicting patient patterns in veterans administration emergency departments. West. J. Emerg. Med..

[35] Bean A.G., Talaga J. (1995). Predicting appointment breaking. J. Health Care Mark..

[36] United States Department of Veterans Affairs (2011). VetPOP 2011.

[37] Siminoff L.A., Hausmann L.R.M., Ibrahim S. (2008). Barriers to obtaining diagnostic testing for coronary artery cisease among veterans. Am. J. Public Health..

